# P-1121. Unconventional Selection Pressures for Resistance in Immunocompromised Gut Resistomes: A Multicenter Observational Study of Pediatric Hematopoietic Stem Cell Transplant Recipients

**DOI:** 10.1093/ofid/ofae631.1308

**Published:** 2025-01-29

**Authors:** Marygrace Duggar, Qidong Jia, Yilun Sun, Li Tang, Ellie Margolis

**Affiliations:** St. Jude Children's Research Hospital, Memphis, Tennessee; St. Jude Children's Research Hospital, Memphis, Tennessee; St. Jude Children's Research Hospital, Memphis, Tennessee; St. Jude Children's Research Hospital, Memphis, Tennessee; St. Jude Children's Research Hospital, Memphis, Tennessee

## Abstract

**Background:**

Increased antibiotic exposure contributes to antibiotic resistance in immunocompromised hosts. Studying how antibiotic courses alter resistance development in their gut community, a common source of bloodstream infections, helps identify treatments posing risk for colonization with Multidrug Resistant Organisms (MDROs).

**Figure:**
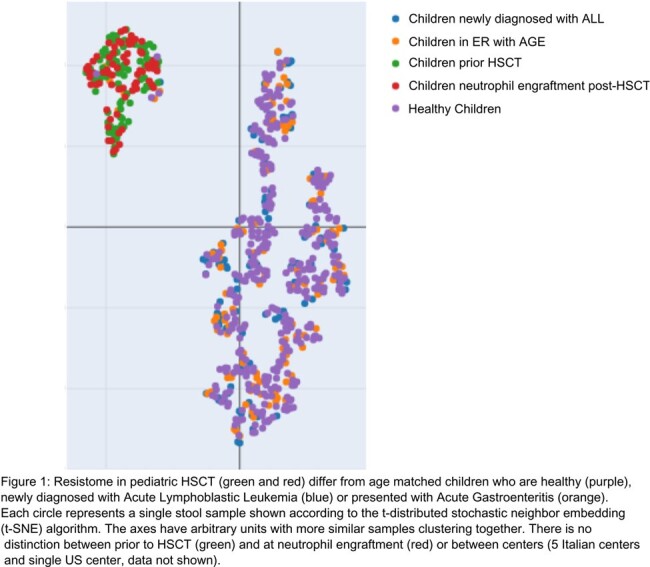

**Methods:**

Stool samples were collected from 163 children pre, day +30, and post-neutrophil engraftment during HSCT, approved by IRBs at St. Jude Children’s Research Hospital and University of Bologna. DNA extracted from these samples underwent Illumina metagenome sequencing, yielding 25 million reads/sample. Antibiotic resistance genes (ARGs) were identified using MEGARes (v 3.0). The children, with median age 8.4, mainly underwent HSCT for Acute Myeloid Leukemia (60%), with majority receiving reduced intensity conditioning (66%). They received multiple antibiotic doses (median 42 daily doses) for fever and neutropenia, infection treatment, and prophylaxis (trimethoprim-sulfamethoxazole at all institutions; fluoroquinolones exclusively at St Jude). ARG abundance was adjusted for sequencing depth and gene length, and changes over time were compared based on antibiotic exposure using linear mixed models.

**Results:**

In pediatric HSCT recipients, the resistome, collection of antibiotic resistance genes in the stool microbial community, differed from healthy children and those with leukemia (fig 1). Surprisingly, levels of antibiotic resistance genes for commonly used antibiotics like Glycopeptides, Pseudomonal Cephalosporins, Aminoglycosides and Fluoroquinolones didn’t increase in children treated with those specific antibiotics. Increases in MDRO related genes (e.g. multidrug efflux pumps, ESBL, vanA) were linked to exposure to antibiotics with anaerobic activity.

**Conclusion:**

Commonly used antibiotics may minimally affect pediatric HSCT patient’s resistomes due to limited gut penetration or prior exposures saturating selection. Other factors like turnover, radiation, inflammation, or conditioning may drive ARG selection. Antibiotics with anaerobic activity link to MDRO related genes suggest colonization resistance may hinder MDRO colonization, warranting increased stewardship.

**Disclosures:**

**Ellie Margolis, MD PhD**, ICLR: Stocks/Bonds (Private Company)

